# Correlation between serum 25-hydroxyvitamin D levels and peripheral neuropathy in type 2 diabetes mellitus

**DOI:** 10.3389/fendo.2025.1541388

**Published:** 2025-06-09

**Authors:** Senzhen Chen, Jinfeng Chen, Shujing Zheng, Yiling Yan, Mengting Huang, Nuoqi Chen

**Affiliations:** ^1^ Zhangzhou Affiliated Hospital of Fujian Medical University, The Second Department of Endocrinology and Metabolism, Zhangzhou, Fujian, China; ^2^ Zhangzhou Affiliated Hospital of Fujian Medical University, Department of Pathology, Zhangzhou, Fujian, China; ^3^ Zhangzhou Affiliated Hospital of Fujian Medical University, Department of Clinical Nutrition, Zhangzhou, Fujian, China; ^4^ Fujian Medical University, Department of Clinical Medicine, Fuzhou, Fujian, China

**Keywords:** serum 25-hydroxyvitamin D, liquid chromatography-tandem mass spectrometry (LC-MS/MS), type 2 diabetes mellitus, DPN, NDPN

## Abstract

**Objective:**

To investigate the correlation between serum 25-hydroxyvitamin D (25[OH]D) levels and diabetic peripheral neuropathy (DPN) in type 2 diabetes mellitus (T2DM) and to evaluate its predictive value for DPN.

**Methods:**

Participants were divided into three groups based on 25(OH)D levels: deficient, insufficient, and normal. The relationship between 25(OH)D and DPN, as well as the correlation of 25(OH)D and DPN with various indicators, was analyzed.

**Results:**

Compared to the non-DPN group (NDPN), the DPN group had significantly lower levels of 25(OH)D3 (22.10 ± 0.77 vs. 24.45 ± 0.66 ng/mL) and total 25(OH)D (23.12 ± 0.74 vs. 25.68 ± 0.67 ng/mL). In addition, significant differences were observed in body mass index (BMI), resting heart rate (RHR), triglyceride (TG), free triiodothyronine (FT3), and blood urea nitrogen (BUN) between the two groups (*p* < 0.05). Multivariate analysis identified 25(OH)D3, total 25(OH)D, FT3, BMI, and RHR as risk factors for DPN. The receiver operating characteristic (ROC) curve analysis revealed that the optimal cutoff value for 25(OH)D3 in predicting DPN in T2DM was 18.85 ng/mL [area under the curve (AUC) 0.76, 95% confidence interval (CI): 0.697–8.823], while the optimal cutoff for total 25(OH)D was 19.94 ng/mL (AUC 0.765, 95% CI: 0.703–0.828).

**Conclusion:**

Serum 25(OH)D levels can serve as a simple and effective screening tool to predict the occurrence of DPN in patients with T2DM.

## 1 Introduction

Diabetes mellitus (DM) is a chronic disease. According to the International Diabetes Federation (IDF), approximately 366 million people worldwide were living with DM in 2021, and this number is expected to rise rapidly to 783 million by 2045 (1). In China, the prevalence of DM has increased rapidly over the past few decades, rising from 0.67% in the 1980s to 12.8% in 2017, affecting an estimated 130 million people. This represents the highest prevalence rate of DM globally, making it a public health issue in China (2). Diabetic peripheral neuropathy (DPN) is one of the most common chronic complications of type 2 diabetes mellitus (T2DM), affecting >90% of patients. Its clinical manifestations range from mild paresthesia to severe conditions such as ulcers, infections, muscle atrophy, neuralgia, neurodegenerative fractures, and Charcot joint disease. DPN is a major risk factor for foot ulcers, infection, gangrene, and amputations. As DPN advances, it diminishes patients’ capacity to work, significantly lowers their quality of daily life, and imposes a considerable financial strain on individuals and families.

Vitamin D, a steroidal hormone, exists primarily in two forms: vitamin D2 (ergocalciferol) and vitamin D3 (cholecalciferol). Previous studies have identified nuclear receptors for vitamin D in neurons and glial cells, suggesting its role in the synthesis of neurotrophic factors and enzymes (3). These findings indicate that vitamin D may delay the progression of DPN through multiple mechanisms. Reduced levels of vitamin D have been associated with an increased risk of DPN in patients with diabetes. A large-scale clinical study conducted abroad revealed that approximately 81% of patients with diabetes have vitamin D deficiency, which is closely associated with the development of DPN (4).

This study aimed to further explore the correlation between serum 25(OH)D levels and DPN in patients with T2DM. By analyzing these relationships, we hope to provide valuable insights for the prevention and treatment of DPN in clinical practice.

## 2 Research objectives and methods

### 2.1 Study participants

Clinical data were collected from patients treated in the Second Department of Endocrinology and Metabolism at Zhangzhou Hospital, Fujian Province, between January 1, 2022, and November 30, 2022. All patients met the 1999 World Health Organization (WHO) diagnostic criteria (5) for T2DM. The exclusion criteria were as follows: 1) acute complications of DM, severe diabetic foot, or acute or chronic inflammatory diseases; 2) other types of DM, including type 1 DM, special types of DM, and gestational DM; 3) comorbidities affecting neuropathy, such as severe spinal disease, severe liver or kidney disease, serious tumors, genetic or metabolic diseases, nutritional deficiencies, connective tissue diseases, trauma, or neuromuscular diseases; 4) recent use of medications known to cause peripheral nerve damage, such as furazolidone or isoniazid, a long history of alcohol consumption, or exposure to toxic substances (such as pesticides and heavy metals); 5) severe primary diseases of the respiratory, circulatory, and hematopoietic system exist; 6) mental illness; and 7) incomplete data collection. Ultimately, 210 participants were included in the study. This study was reviewed and approved by the ethics committee of our hospital.

### 2.2 Method

General data collection: Clinical information on the study participants was collected, including sex, age, height, weight, waist circumference, duration of diabetes, diabetes complications, hypertension, past medical history, smoking history, drinking history, and body mass index (BMI).

Detection of clinical indicators: All study participants fasted for 8 h, and peripheral venous blood samples were collected by professional medical staff in the early morning of the following day. The following indicators were measured: serum 25(OH)D, glycated hemoglobin (HbA1c), fasting C peptide, total cholesterol (TC), triglyceride (TG), high-density lipoprotein cholesterol (HDL-C), low-density lipoprotein cholesterol (LDL-C), blood urea nitrogen (BUN), serum uric acid (SUA), blood phosphorus, blood calcium, full parathyroid hormone (PTH), free triiodothyronine (FT3), free thyroxine (FT4), thyroid-stimulating hormone (TSH), and other indicators.

Determination of serum 25(OH)D: Serum 25(OH)D levels were measured using liquid chromatography–tandem mass spectrometry (LC–MS/MS).

Study groups: Participants were stratified into three groups based on serum 25(OH)D levels, following the “Consensus on the clinical application of vitamin D and its analogs”. Group A (deficiency group) included individuals with serum 25(OH)D levels <20 ng/mL (51 cases). Group B (insufficient group) included those with levels between 20 and 30 ng/mL (112 cases). Group C (normal group) included individuals with levels ≥30 ng/mL (47 cases). For the diagnosis of DPN, the study adhered to the Guidelines for the Prevention and Treatment of Type 2 Diabetes (2017 edition, China). The diagnostic criteria were as follows: 1) a clear history of diabetes; 2) neuropathy occurring at or after the diagnosis of diabetes; 3) clinical symptoms of neuropathy, such as pain, numbness, or paresthesia assessed using five tests (ankle reflex, vibration, pressure, temperature, and pinprick sensation; and 4) if no clinical symptoms were present, abnormalities in any two of the five tests could confirm the diagnosis and exclusion of neuropathy caused by other factors. If the diagnosis remained uncertain, neuroelectrophysiological examinations were performed for differential diagnosis. Based on these criteria, the 210 participants were divided into two groups: the non-DPN (NDPN) group (100 cases) and the DPN group (110 cases).

### 2.3 Statistical methods

Statistical analysis was performed using the SPSS 27.0 software. The normality of the distribution for measurement data was tested using the Shapiro–Wilk test. Normally distributed data were described using mean ± standard deviation (x ± s) and analyzed using the t-test for two groups or one-way analysis of variance for multiple groups. Non-normally distributed data were described using the median and interquartile range (M [P25, P75]) and analyzed using the non-parametric Mann–Whitney U test for two groups or the Kruskal–Wallis H test for multiple groups. Categorical data were described as frequencies and percentages [n (%)] and analyzed using the chi-square (χ^2^) test for two groups or pairwise comparisons for multiple groups. Spearman’s rank correlation analysis was used to assess the relationship between clinical data, 25(OH)D levels, and DPN. Ordered logistic regression was performed to analyze the association between 25(OH)D levels and multiple influencing factors. Univariate and multivariate binary logistic regression analyses were conducted to identify the main risk factors of DPN. The receiver operating characteristic (ROC) curve was used to determine the optimal cutoff value for the main risk factors based on the maximum Youden index. The clinical predictive value was assessed using the area under the ROC curve. All statistical tests were two-sided at *p* < 0.05 and a highly significant level at *p* < 0.01.

## 3 Results

### 3.1 Intergroup analysis of different serum 25-hydroxyvitamin D groups

#### 3.1.1 Comparison of general clinical data among different serum 25-hydroxyvitamin D level groups

A comparison of general clinical data between groups A, B, and C revealed significant differences in blood calcium (Ca) and DPN (*p* < 0.05). However, no significant differences were observed in sex, age, resting heart rate (RHR), waist circumference, BMI, hypertension (HBP), nocturnal hypoglycemia, HbA1c, blood phosphorus (P), TG, TC, HDL-C, LDL-C, BUN, TSH, FT3, FT4, fasting C-peptide, UA, PTH, peripheral arterial disease (PAD), diabetic kidney disease (DKD), and diabetic retinopathy (DR) (*p* > 0.05) (**Table 1**).

**Table 1:** Comparing common data across subgroups of vitamin D levels.

Factors	Group A (n = 51)	Group B (n = 112)	Group C (n = 47)	F/z/χ^2^	*p*
Sex (M/F)	(30/21)	(68/44)	(24/23)	1.281^a^	0.527
Age (year)	52.24 ± 13.88	57.02 ± 11.11	58.26 ± 11.80	0.274^c^	0.761
RHR (sub/min)	87 (78, 97)	84 (78, 95)	81.5 (74, 88)	3.16^d^	0.206
Waistline (cm)	86 (21.51, 25.26)	85 (21.51, 25.26)	85 (21.51, 25.26)	0.717^d^	0.410
BMI (kg/m^2^)	23.43 (22.30, 25.20)	23.47 (21.48, 24.78)	23.62 (22.22, 25.39)	0.420^d^	0. 811
HBP (%)	27 (52.9%)	45 (40.2%)	26 (55.3%)	3.665^a^	0.160
Hypoglycemia (%)	9 (17.6%)	12 (10.7%)	2 (4.3%)	4.34 1^a^	0.114
HbA1c (%)	10.35 (7.72, 12.23)	9.42 (7.72, 11.54)	12.29 (8.43, 12.48)	0.037^d^	0.982
P (mmol/L)	1.30 (1.15, 1.48)	1.27 (1.12, 1.49)	1.26 (1.09, 1.49)	2.073^d^	0.355
Ca (mmol/L)	2.22 (2.17, 2.29)	2.31 (2.23, 2.38)	2.32 (2.22, 2.39)	10.71^d^	0.005*
TG (mmol/L)	1.26 (1.01, 1.72)	1.43 (1.04, 2.48)	1.85 (1.42, 2.48)	4.404^d^	0.111
TC (mmol/L)	4.72 (3.72, 5.56)	4.78 (4.10, 5.72)	4.86 (3.86, 5.59)	1.396^d^	0.498
HDL-C (mmol/L)	1.03 (0.89, 1.24)	1.09 (0.91, 1.28)	0.93 (0.86, 1.10)	2.043^d^	0.360
BUN (mmol/L)	5.50 (4.30, 7.40)	5.4 (4.70, 6.60)	5.50 (4.50, 7.30)	0.216^d^	0.898
TSH (pmol/L)	1.55 (1.13, 2.25)	1.38 (0.79, 2.11)	1.71 (0.94, 2.73)	2.529^d^	0.282
FT3 (pmol/L)	4.72 (4.30, 5.18)	4.85 (4.40, 5.30)	4.94 (4.41, 5.40)	1.522^d^	0.467
FT4 (pmol/L)	11.60 (10.10, 13.3)	11.70 (10.8, 13.45)	11.65 (10.2, 14.80)	0.703^d^	0.703
Fasting C-peptide (ng/mL)	0.76 (0.50, 1.06)	0.77 (0.56, 1.32)	1.11 (0 63, 1.69)	2.600^d^	0.273
UA (μmol/L)	311.22 ± 112.22	323.97 ± 90.63	323.21 ± 126.59	0. 275^c^	0.76
PTH (pg/mL)	41.3 (32.30, 49.90)	33.90 (27.05, 41.60)	37.05 (23.30, 52.70)	2.462^d^	0.297
DPN (%)	36 (70.6%)	54 (47.8%)	20 (42.6%)	9.378^a^	0.009*
PAD (%)	30 (58.8%)	72 (64.3%)	28 (59.6%)	0.089^a^	0.956
DKD (%)	11 (21.6%)	22 (19.6%)	12 (25.5%)	0.857^a^	0.651
DR (%)	13 (25.5%)	21 (18.6%)	10 (21.3%)	1.149^a^	0.563

Data are expressed as mean ± standard deviation, median (interquartile range), and number of cases (%).

RHR, resting heart rate; BMI, body mass index; HBP, hypertension; TG, triglyceride; TC, total cholesterol; HDL-C, high-density lipoprotein cholesterol; BUN, blood urea nitrogen; TSH, thyroid-stimulating hormone; FT3, free triiodothyronine; FT4, free thyroxine; PTH, parathyroid hormone; DPN, diabetic peripheral neuropathy; PAD, peripheral arterial disease; DKD, diabetic kidney disease; DR, diabetic retinopathy.

a is χ^2^ values.

c is the F value.

d is the z-value.

*p < 0.05, indicating a statistically significant difference.

#### 3.1.2 Correlation analysis of serum 25 hydroxyvitamin D and various factors

Based on the comparison of different serum 25-hydroxyvitamin D levels with general clinical, laboratory, and characteristic data, the correlations between serum 25-hydroxyvitamin D and the statistically significant factors were analyzed. The results showed that serum 25-hydroxyvitamin D was positively correlated with blood calcium levels (r = 0.224, *p* < 0.0.01) and negatively correlated with DPN (r = −0.0195, *p* < 0.005) (*p* < 0.05). These findings suggest a strong association between serum 25-hydroxyvitamin D and DPN. Further details are presented in **Table 2**.

**Table 2:** Analysis of the association between vitamin D and various factors.

Factor	r	*p*
DPN (case %)	−0.195	0.005*
Ca (mmol/L)	0.224	0.001*

r denotes the correlation coefficient.

DPN, diabetic peripheral neuropathy; Ca, blood calcium.

*p < 0.05, indicating a statistically significant difference.

#### 3.1.3 Ordered logistic regression analysis of 25(OH)D3 and related influencing factors

Using different 25(OH)D levels as the dependent variables, a logistic regression analysis was performed with Ca and DPN as the independent variables. The final retained factors were Ca and DPN (*p* < 0.05), indicating that DPN [odds ratio (OR) = 1.100, 95% confidence interval (CI): 0.224–1.301, *p* = 0.006] and Ca (OR = 1.048, 95% CI: 0.486–3.755, *p* = 0.011) were independently associated with 25(OH)D (**Table 3**).

**Table 3:** Ordered logistic regression analysis of different vitamin D subgroups.

Correlative factor	β	SE	χ^2^	*p*	OR (95% CI)
DPN	0.763	0.275	7.691	0.006*	1.100 (0.224, 1.301)
Ca (mmol/L)	2.120	0. 834	6.491	0.011*	1.048 (0.486, 3.755)

OR, odds ratio; 95% CI, 95% confidence interval; DPN, diabetic peripheral neuropathy; Ca, blood calcium.

*p < 0.05, indicating a statistically significant difference.

### 3.2 Intergroup analysis between the NDPN and DPN groups

#### 3.2.1 Comparison of general data between the NDPN and DPN groups

A comparison of the general data between the NDPN and DPN groups revealed statistically significant differences in BMI, RHR, TG, FT3, BUN, 25(OH)D3, total 25(OH)D (*p* < 0.05), sex, age, HBP, hypoglycemia, HbA1c, P, Ca, TC, HDL-C, LDL-C, TSH, FT4, PTH, fasting C-peptide, and UA (*p* > 0.05). Both BMI and RHR were higher in the DPN group than in the NDPN group (**Table 4**).

**Table 4:** Comparison of common data between NDPN and DPN.

Factors	NDPN (n = 1 00)	DPN (n = 110)	χ^2^/t/z	*p*
Sex (M/F)	(59/41)	(63/47)	0.64^a^	0.800
Age (year)	55.51 ± 11.55	59.48 ± 12.03	−2.29^b^	0.819
BMI (kg/m^2^)	23.11 (21.50, 24.74)	23.61 (21.80, 25.26)	−4.562^d^	<0.001*
RHR (sub/min)	83 (77, 93)	85.5 (78, 97)	−4.562^d^	<0.001*
HBP (%)	54 (54%)	56 (50.9%)	0.201^a^	0.654
Hypoglycemia (%)	7 (6%)	16 (14.5%)	3.085^a^	0.08
HbA1c (%)	9.54 (8.10, 11.92)	10.09 (7 68, 12.07)	−1.191^d^	0.234
P (mmol/L)	1.26 (1.1, 1.37)	1.28 (1.12, 1.51)	−1.070^d^	0.285
Ca (mmol/L)	2.29 (2.20, 2.36)	2.29 (2.20, 2.35)	−0.098^d^	0.922
TG (mmol/L)	1. 64 (1.25, 2.30)	1. 26 (0.98, 1.8 1)	−2.360^d^	0.018*
TC (mmol/L)	4.92 (4.21, 5.67)	4.64 (3.78, 5.56)	−1.072^d^	0.284
HDL-C (mmol/L)	0.98 (0.86, 1.20)	1.07 (0.93, 1.96)	−1.487^d^	0.137
LDL-C (mmol/L)	3.22 (2.68, 3.60)	3.03 (2.18, 3.73)	−0.982^d^	0.326
TSH (pmol/L)	1.51 (0.79, 2.18)	1.45 (1.00, 2.52)	−0.924^d^	0.924
FT3 (pmol/L)	4.96 (4.54, 5.38)	4.72 (4.27, 5.19)	−0.005^d^	0.005*
FT4 (pmol/L)	11.60 (10.80, 13.80)	11.70 (10.10, 13.50)	−0.826^d^	0.826
PTH (pg/mL)	37.20 (28.30, 49.40)	33.40 (28.10, 44.80)	−1.330^d^	0.183
BUN (mmol/L)	5.30 (4.20, 6.10)	6.05 (4.90, 7.90)	−10.280^d^	<0.001*
Fasting C-peptide (ng/mL)	0.87 (0.63, 1.18)	0.81 (0.55, 1.31)	−0.932	0.351
UA (μmol/L)	322.5 ± 8.77	311.2 ± 11.29	5.92^b^	0.783
25(OH)D2 (ng/mL)	0.545 (0.290, 0.960)	0.445 (0.120, 0.930)	−0.702^b^	0.438
25(OH)D3 (ng/mL)	24.45 ± 0. 66	22.10 ± 0.77	1.532^b^	0.026*
Total 25(OH)D (ng/mL)	25.68 ± 0.67	23.12 ± 0.74	0.657^b^	0.037*

Data are expressed as mean ± standard deviation, median (interquartile range), and number of cases (%).

NDPN, non-diabetic peripheral neuropathy; DPN, diabetic peripheral neuropathy; BMI, body mass index; RHR, resting heart rate; HBP, hypertension; HbA1c, glycated hemoglobin; Ca, blood calcium; TG, triglyceride; TC, total cholesterol; HDL-C, high-density lipoprotein cholesterol; LDL-C, low-density lipoprotein cholesterol; TSH, thyroid-stimulating hormone; FT3, free triiodothyronine; FT4, free thyroxine; PTH, parathyroid hormone; BUN, blood urea nitrogen; UA, uric acid.

a is χ^2^.

b is the t.

d is the z.

*p < 0.05, indicating a statistically significant difference.

#### 3.2.2 Correlation analysis between DPN and various factors

Based on the comparison of general data, laboratory data, 25(OH)D levels, and other factors between the NDPN and DPN groups, an analysis of correlations with DPN was conducted. The results indicated that DPN was positively correlated with BUN (r = 0.869, *p* < 0.001) and RHR (r = 0.579, *p* < 0.001). Conversely, DPN negatively correlated with 25(OH)D3 (r = −0.192, *p* = 0.005), total 25(OH)D (r = −0.192, *p* = 0.005), FT3 (r = −0.202, *p* = 0.004), TG (r = −0.164, *p* = 0.018), and BMI (r = −0.709, *p* < 0.001). Among these, DPN showed a strong correlation with 25(OH)D3, total 25(OH)D, FT3, BMI, BUN, and RHR (**Table 5**).

**Table 5:** Association analysis of DPN and related factors.

Factors	r	*p*
25(OH)D3 (ng/mL)	−0.192	0.005*
Total 25(OH)D (ng/mL)	−0.192	0.005*
FT3 (pmol/L)	−0.202	0.004*
TG (mmol/L)	−0.164	0.018*
BUN (mmol/L)	0.869	<0.001*
BMI (kg/m^2^)	−0.709	<0.001*
RHR (sub/min)	0.579	<0.001*

r denotes the correlation coefficient.

DPN, diabetic peripheral neuropathy; FT3, free triiodothyronine; TG, triglyceride; BUN, blood urea nitrogen; BMI, body mass index; RHR, resting heart rate.

*p < 0.05, indicating a statistically significant difference.

#### 3.2.3 Logistic regression analysis of DPN and influencing factors

Univariate binary logistic regression analysis was conducted, with the occurrence of DPN as the dependent variable and various clinical indicators as the independent variables. The results showed that 25(OH)D3 (OR = 0.944, 95% CI: 0.908–0.981, *p* = 0.003), total 25(OH)D (OR = 0.944, 95% CI: 0.907–0.981, *p* = 0.004), FT3 (OR = 0.566, 95% CI: 0.379–0.845, *p* = 0.005), BMI (OR = 0.119, 95% CI: 0.70–0.200, *p* < 0.001), and RHR (OR = 1.160, 95% CI: 1.115–1.206, *p* < 0.001) may serve as risk factors for predicting the occurrence of DPN (*p* < 0.05) (**Table 6**).

**Table 6:** Univariate binary logistic regression analysis of DPN.

Factors	β	SE	Wald	*p*	OR (95% CI)
25(OH)D (ng/mL)	−0.58	0.20	8.601	0.003*	0.944 (0.908, 0.981)
Total 25(OH)D (ng/mL)	−0.58	0.20	8.377	0.004*	0.944 (0.907, 0.981)
FT3 (pmol/L)	−0.569	0.205	7.729	0.005*	0.566 (0.379, 0.845)
TG (mmol/L)	−0.257	0.132	3.796	0.051	0.774 (0.598, 1.002)
BMI (kg/m^2^)	−2.132	0.267	63.596	<0.001*	0.119 (0.70, 0.200)
RHR (sub/min)	0.148	0.20	55.115	<0.001*	1.160 (1.115, 1.206)

OR, odds ratio; 95% CI, 95% confidence interval; DPN, diabetic peripheral neuropathy; FT3, free triiodothyronine; TG, triglyceride; BMI, body mass index; RHR, resting heart rate.

*p < 0.05, indicating a statistically significant difference.

Using the occurrence of DPN as the dependent variable and the identified risk factors as the independent variables, a multivariate binary logistic regression model was established. After adjusting for confounding factors, the results indicated that total 25(OH)D (OR = 0.935, 95% CI: 0.893–0.978, *p* < 0.004) and FT3 (OR = 0.590, 95% CI: 0.387–0.900, *p* = 0.014) were protective factors against DPN (*p* < 0.05) (**Table 7**).

**Table 7:** Multivariate binary logistic regression analysis of DPN.

Factors	β	SE	Wald	*p*	OR (95% CI)
Total 25(OH)D (ng/mL)	−0.067	0.023	8.492	0.004*	0.935 (0.893, 0.978)
FT3 (pmol/L)	−0.527	0.216	5.985	0.014*	0.590 (0.387, 0.900)
BMI (kg/m^2^)	0.008	0.019	0.186	0.666	1.008 (0.971, 1.046)
RHR (sub/min)	0.019	0.011	2.806	0.094	1.019 (0.997, 1.041)

DPN, diabetic peripheral neuropathy; FT3, free triiodothyronine; BMI, body mass index; RHR, resting heart rate.

*p < 0.05, indicating a statistically significant difference.

Taking the occurrence of DPN as the dependent variable and the identified risk factors as the independent variables, a multivariate binary logistic regression model was established. After adjusting for confounding factors, the results showed that 25(OH)D3 (OR = 0.944, 95% CI: 0.906–0.984, *p* < 0.007) and FT3 (OR = 0.593, 95% CI: 0.388–0.905, *p* = 0.015) were protective factors against DPN (*p* < 0.05) (**Table 8**).

**Table 8:** Multivariate binary logistic regression analysis of DPN.

Factors	β	SE	Wald	*p*	OR (95% CI)
25(OH)D3 (ng/mL)	−0.057	0.021	7.302	0.007*	0.944 (0.906, 0.984)
FT3 (pmol/L)	−0. 523	0.216	5.865	0.015*	0.593 (0.388, 0.905)
BMI (kg/m^2^)	0.008	0.019	0.198	0.656	1.008 (0.972, 1.046)
RHR (sub/min)	0.019	0.11	2.975	0.085	1.020 (0.997, 1.042)

DPN, diabetic peripheral neuropathy; FT3, free triiodothyronine; BMI, body mass index; RHR, resting heart rate.

*p < 0.05, indicating a statistically significant difference.

#### 3.2.4 ROC curve analysis

**Figure 1:**
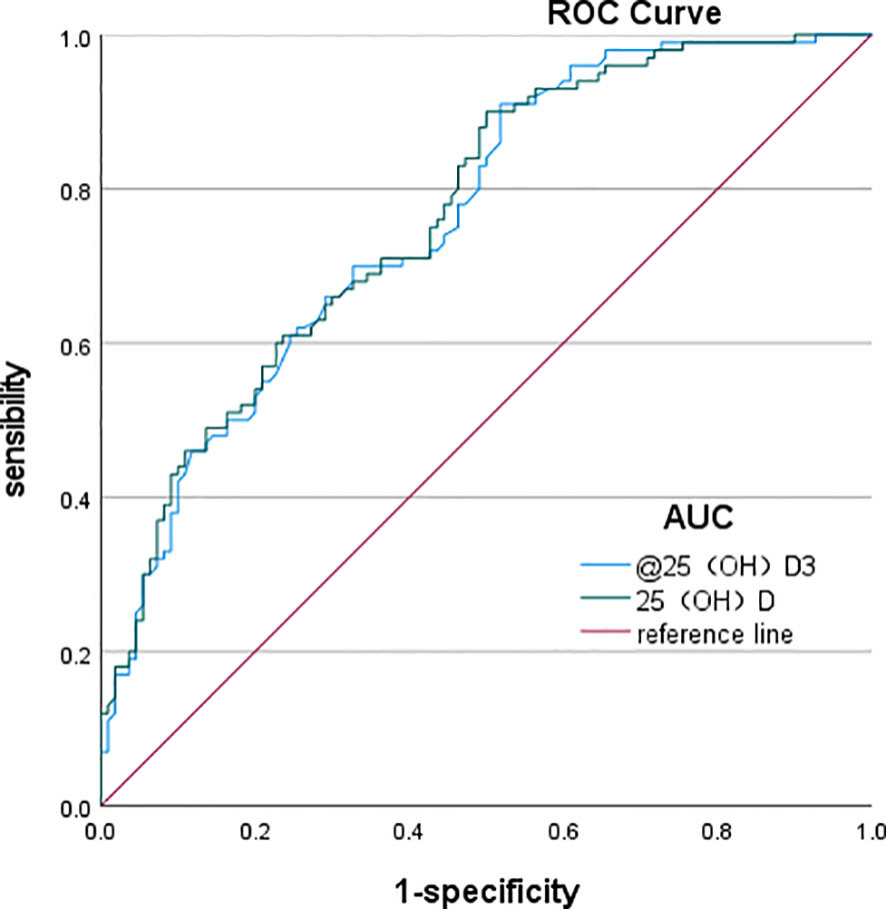
ROC curves of total 25(OH)D and 25(OH)D3 predicting the occurrence of DPN. ROC, receiver operating characteristic; DPN, diabetic peripheral neuropathy.

The ROC curve analysis revealed that the optimal cutoff value of 25(OH)D3 for predicting DPN in T2DM was 18.85 ng/mL, with an area under the curve (AUC) of 0.76, sensitivity of 91%, and specificity of 48.2%. Similarly, the optimal cutoff value of 25(OH)D for predicting T2DM was 19.94 ng/mL, with an AUC of 0.765, a sensitivity of 90%, and a specificity of 50% (**Table 9**, **Figure 1**).

**Table 9:** ROC curve analysis of 25(OH)D3 and 25(OH)D in predicting DPN.

Factors	AUC	*p*	95% CI		Cutoff value	Sensitivity	Specificity	Youden’s index
25(OH)D3	0.76	<0.001*	0.697	0.823	18.85	91%	48.2%	0.392
25(OH)D	0.765	<0.001*	0.703	0.828	19.94	90%	50.0%	0.4

ROC, receiver operating characteristic; DPN, diabetic peripheral neuropathy; AUC, area under the curve.

*p < 0.05, indicating a statistically significant difference.

## 4 Discussion

The incidence of T2DM, a complex metabolic disease affecting both developing and developed countries, has been reported to be increasing at an alarming rate (6). With rapid economic development, industrialization, lifestyle changes, and an aging population, the prevalence of diabetes in China has risen rapidly. It has become a major chronic non-communicable disease, ranking among the leading health threats alongside cardiovascular diseases and cancer. According to the WHO, China’s economic losses due to diabetes and related cardiovascular diseases amounted to $557.7 billion between 2005 and 2015 (7). Globally, the prevalence of diabetes has increased dramatically, reaching 8.3% in 2014, which corresponds to approximately 3.87 million cases (8). In the early stages, diabetes may present with frequent symptoms such as dry mouth, polydipsia, polyuria, or weight loss, which often do not affect daily life and may be overlooked. However, as the disease progresses, poor blood glucose control and long-term metabolic disorders can lead to chronic microvascular complications, including nerve, eye, and kidney damage, as well as an increased risk of cardiovascular and cerebrovascular diseases. Diabetic microvascular complications, such as diabetic neuropathy, retinopathy, and nephropathy, typically occur in patients who have had diabetes for several years or even decades (9). T2DM is associated with systemic chronic inflammation, characterized by increased concentrations of acute-phase response proteins and inflammatory markers. These inflammatory mediators contribute to insulin resistance and β-cell dysfunction (10, 11). Since vitamin D possesses anti-inflammatory and immunomodulatory properties, it can help alleviate low-grade chronic inflammation in diabetes by regulating cytokine production (12). The main pathogenesis of T2DM involves insulin resistance. Vitamin D not only enhances insulin secretion by regulating insulin receptor gene expression but also promotes the repair of damaged islet cells and stimulates β-cell function, thereby improving insulin sensitivity and glycemic control of patients with T2DM (5). Therefore, vitamin D has a protective effect against T2DM, and vitamin D deficiency can lead to an increased risk of T2DM (6).

DPN is one of the most common chronic complications of DM, affecting a wide range of patients with varying degrees of severity (13). There is usually an increased risk of physical disability, cardiovascular disease, and mortality. At least 20% of patients with type 1 diabetes develop distal symmetric polyneuropathy within 20 years, while 10% to 20% of newly diagnosed type 2 diabetic patients develop the condition, with prevalence rising to 50% within 10 years. The neurological complications associated with DPN place a huge burden on both the patient and society (14). DPN mainly leads to decreased quality of life due to pain, sensory loss, gait instability, foot ulceration, and amputation (15). Furthermore, its progression can lead to vision loss, neurological impairment, reduced mobility and cognitive function, decreased quality of life, limited employment, lower productivity, and increased healthcare costs. Without treatment, irreversible damage or even death may occur (16). DPN development has been attributed to various possible etiologies. Over the past few decades, it has primarily been believed that prolonged hyperglycemia leads to several damaging mechanisms, including the activation of the polyol pathway, the generation of oxygen radicals, the formation of advanced glycation end products, inflammation, impaired microcirculation, oxidative stress, and both direct and indirect nerve damage. However, recent studies have suggested that, in addition to glycemic factors, factors such as age-related neuronal degradation, hypertension, blood lipid levels, body weight, and decreased neurotrophic factors interact, leading to the occurrence and development of peripheral neuropathy. Later studies have found that vitamin D levels were substantially reduced in patients with DPN. It has been suggested that vitamin D deficiency is a risk factor for DPN and may be involved in its development, such as in oxidative stress caused by hyperglycemia, inflammation, and neuronal ischemia. The mechanism of DPN is still not completely understood, and in addition to strict glycemic control, effective modified therapy for DPN is lacking (17, 18). The identification of DPN risk factors is crucial for a better understanding of the mechanisms underlying DPN and for more effective treatments.

Vitamin D, like vitamins A, E, and K, is a fat-soluble vitamin that exists in two forms: vitamin D3 (cholecalciferol) and vitamin D2 (ergocalciferol). Vitamin D3 is synthesized in the skin upon exposure to ultraviolet B (UV-B) irradiation, while vitamin D2 is obtained from dietary sources and converted to ergocalciferol under UV irradiation. In the liver, both cholecalciferol and ergocalciferol are hydroxylated to form 25(OH)D2 and 25(OH)D3. These metabolites are further converted in the kidneys by 1-α-hydroxylase into the biologically active form, 1.25(OH)D (19). Among these forms, 25(OH)D is the most abundant vitamin D metabolite in circulation, serving as the primary storage form in the body. Due to its long plasma half-life and stability, 25(OH)D is considered the gold standard for assessing serum vitamin D levels in clinical studies (20). In this study, 25(OH)D levels were measured using LC–MS/MS. The study examined serum 25(OH)D, serum 25(OH)D3, and serum 25(OH)D2, with 25(OH)D being the primary study index.

In addition to participating in bone metabolism, vitamin D is also involved in the pathogenesis of various diseases, including cardiovascular diseases, metabolic diseases, cancers, multiple sclerosis, biological infections, and autoimmune conditions (21). It also plays a key role in the proliferation and differentiation of immune cells and other immune regulations of the body (22). Vitamin D deficiency is widespread, affecting 30%–87% of the population. 25(OH)D concentration between 20 and 30– ng/mL (50–75 nmol/L) is considered insufficient, while levels below 20 ng/mL (<50 nmol/L) indicate vitamin D deficiency. Vitamin D also plays a critical role in insulin secretion by binding to vitamin D receptors expressed on pancreatic β-cells (23). It enhances insulin sensitivity by binding to the insulin receptor expression by binding to the vitamin D response element present in the promoter of the human insulin receptor gene (23, 24). It also influences fatty acid metabolism in insulin-responsive tissues by activating transcription factors (25) and protects against cytokine-induced apoptosis (26–28). Therefore, there is an inverse relationship between vitamin D levels and the risk of diabetic complications.

To investigate the relationship between DPN and 25(OH)D, this study categorized patients with T2DM into the DPN and NDPN groups based on diagnostic criteria. A comparison of general data and biochemical indicators between the two groups revealed that 25(OH)D and 25(OH)D3 levels were reduced in both groups. However, the mean 25(OH)D level in the DPN group (23.12 ± 0.74 ng/mL) was significantly lower than that in the NDPN group (25.68 ± 0.67 ng/mL). Similarly, the mean 25(OH)D3 level in the DPN group (22.10 ± 0.77 ng/mL) was significantly lower than that in the NDPN group (24.45 ± 0.66 ng/mL). In addition, 81.8% of patients in the DPN group exhibited reduced 25(OH)D and 25(OH)D3 levels, compared to 73% in the NDPN group, indicating that 25(OH)D reduction is more prevalent in DPN. An observational study on patients with T2DM aged >5 years demonstrated a significant reduction in 25(OH)D levels. This suggests that lower 25(OH)D levels serve not only as a relevant risk factor for DPN but also as a new therapeutic option for DPN. Furthermore, studies have shown that vitamin D levels are negatively correlated with the presence and severity of DPN. The severity of nerve conduction velocity decreased as vitamin D levels increased. After 3 months of vitamin D supplementation, neuropathic pain symptoms were reduced by approximately 50% (29). However, some scholars have raised doubts. Therefore, there is still controversy about whether to supplement vitamin D and what is its best dosage and course of treatment, which need to be verified by larger clinical trials (30).

Serum 25(OH)D levels were further categorized based on “Consensus on the clinical application of vitamin D and its analogs” into group A (deficiency group) with serum 25(OH)D < 20 ng/mL, group B (insufficient group) with levels between 20–30 ng/mL, and group C (normal group) with levels >30 ng/mL; their incidence of peripheral neuropathy was 70.6%, 47.8%, and 42.6%, respectively. As serum 25(OH)D levels increased, the incidence of DPN gradually decreased. Logistic regression analysis was used to assess the effects of 25(OH)D3, 25(OH)D, FT3, TG, BMI, RHR, and BUN on DPN. The results indicated that serum 25(OH)D and FT3 were protective factors against DPN in T2DM.

The ROC curve analysis demonstrated that the optimal cutoff value of 25(OH)D3 for predicting DPN in T2DM was 18.85 ng/mL, yielding an AUC of 0.76. In addition, the optimal cutoff for 25(OH)D for predicting T2DM was 19.94 ng/mL, with 90% sensitivity and 50% specificity, resulting in an AUC of 0.765.

The results indicated that patients with T2DM with lipid disorders (especially TG) are at higher risk of developing DPN. Even without T2DM, hypertriglyceridemia remained significantly associated with peripheral neuropathy. Patients with elevated TG levels were 2.1 times more likely to develop DPN, highlighting it as an independent risk factor, especially for small unmyelinated axons (31). However, in our study, TG levels were higher in the NDPN group, potentially due to poor recent blood glucose control, lipid-lowering medication use, dietary differences, smoking, and alcohol consumption, which may have influenced the results.

In addition, this study identified FT3 as a protective factor against DPN. T3 plays a role in inhibiting sensory neuron axonal expansion while stimulating the formation of new axons, contributing to nerve repair and regeneration. Animal studies have previously shown that T3 promotes dorsal root ganglia and neuron growth. In a chronic hyperglycemic state, reduced T3 levels lead to vascular endothelial dysfunction, resulting in insufficient nerve blood supply and damage to the peripheral nerves. Thyroid hormones protect microvascular endothelial cells, thereby reducing hyperglycemia-induced nerve damage. Furthermore, studies have shown that abnormal thyroid function may affect vitamin D metabolism, and patients with subclinical hypothyroidism often exhibit concurrent vitamin D deficiency, with both factors synergistically increasing the risk of DPN (32).In T2DM patients, the simultaneous evaluation of thyroid function (such as TSH and FT3) and vitamin D levels may help to identify high-risk groups of DPN in the early stage. However, the interaction mechanism between thyroid hormone sensitivity index and vitamin D is not clear, and more studies are needed to confirm it.

### 4.1 Study limitations

This study has some limitations worth noting. First, owing to the retrospective study design, the study did not include a non-diabetic control group. Second, the sample size was small, reducing the generalizability of the findings. Third, no long-term follow-up was conducted to track 25(OH)D levels before and after DPN treatment, making it difficult to assess its role as a therapeutic marker. In addition, the study did not account for dietary intake, calcium supplementation, sunlight exposure, and environmental factors, all of which could influence serum 25(OH)D levels.

## 5 Conclusion

A reduction in 25(OH)D levels in patients with T2DM is closely related to the occurrence of DPN. 25(OH)D serves as a protective factor against DPN, and when serum levels drop below 19.94 ng/mL, the risk of developing DPN increases. Therefore, monitoring 25(OH)D levels may aid in the early diagnosis of DPN and provide a basis for timely intervention.

## Data Availability

The raw data supporting the conclusions of this article will be made available by the authors, without undue reservation.
